# Neuropsychological patterns in systemic lupus erythematosus patients with depression

**DOI:** 10.1186/ar2203

**Published:** 2007-05-15

**Authors:** Elizabeth Kozora, David B Arciniegas, Lening Zhang, Sterling West

**Affiliations:** 1 Department of Medicine, National Jewish Medical and Research Center, 1400 Jackson St., Denver, CO 80206, USA; 2 University of Colorado School of Medicine, 4200 E. 9th Avenue, Denver, CO, 80220, USA; 3 Division of Biostatistics, National Jewish Medical and Research Center, 1400 Jackson Street, Denver, CO, 80206, USA

## Abstract

Thirteen patients with systemic lupus erythematosus and depression (Depressed-SLE), 10 Depressed-Control subjects, and 25 Healthy Control subjects completed cognitive testing and self-report questionnaires of pain, depression, and fatigue. The Depressed-SLE group scored higher on the American College of Rheumatology Neuropsychological Battery for systemic lupus erythematosus cognitive impairment index compared to Depressed-Control and Healthy Control subjects (*p *< 0.05 and *p *< 0.02, respectively). No correlations between cognitive impairment and pain, fatigue, or perceived cognitive failures were observed in the Depressed-SLE participants. Moderate agreement (86.4%) was found between a comprehensive neuropsychology battery cognitive impairment index and the ACR-SLE impairment index in the Depressed-SLE patients. Overall, the magnitude and pattern of cognitive impairment in Depressed-SLE patients cannot be explained by depression alone.

## Introduction

More than 50% of patients with systemic lupus erythematosus (SLE) demonstrate major psychiatric and neurological disorders indicating central nervous system (CNS) involvement [1,2]. Neuropsychiatric manifestations in SLE are diverse and include major manifestations (that is, stroke syndromes, seizures, psychotic episodes, and so on) or less severe abnormalities, including headaches, minor mood disorders, and cognitive difficulties [3]. Depression is the most frequently documented psychiatric problem in patients with SLE [4-7]. However, the role of depression in lupus remains controversial, and it is not known if depression is associated with the effects of a chronic illness or if it represents a manifestation of CNS involvement in this population.

There is some discrepancy in the literature regarding the association between psychological factors and cognitive functions in patients with SLE. Although some studies have demonstrated that patients with SLE with overt neuropsychiatric disorders (NPSLE) have strong correlations between psychological and cognitive distress [8-13], other studies have found no relationship between these factors in NPSLE [14-16]. There is consistency in the literature regarding patients with non-NPSLE (SLE with inactive disease without overt neuropsychiatric disorders); few studies have shown a relationship between cognitive and psychological status and depression in these groups [11,14,16-19]. Thus, despite the strong relationships reported between psychological distress and neurobehavioral factors in some patients with SLE, this relationship remains unclear in others. Additionally, the underlying mechanisms are unclear and pose difficult diagnostic and treatment issues.

Several studies have demonstrated that patients with NPSLE have stronger correlations between psychological and cognitive distress [8-12,20]. In contrast, a few studies found no relationship between psychiatric histories, psychological status, and cognitive status in NPSLE [14-16]; some patients with non-NPSLE show little relationship between cognitive and psychological status and depression [11,16-19]. Thus, despite the strong relationships reported between psychological distress and neurobehavioral factors, the underlying mechanisms are unclear and pose difficult diagnostic and treatment issues.

Denburg and colleagues [21] used questionnaires to classify patients with SLE as having the presence or absence of psychiatric distress and then compared performance on cognitive measures between the two groups. The results indicated that cognitively impaired SLE patients with the presence of psychiatric distress showed greater impairment in the areas of short-term retention and verbal fluency when compared to cognitively impaired SLE patients without psychiatric distress. A study by Hay and colleagues [10] showed that improvement in cognitive abilities paralleled improvement in psychological status as measured by ratings of psychiatric disability through standardized psychiatric interviews. In a study of 101 patients with SLE, Holliday and colleagues [22] reported that age, education, and depression (as measured by a self-report inventory) were strong predictors of neuropsychological test performance. In our recent study [11], we noted that NPSLE participants had strong correlations between self-reported measures of depression and cognitive summary scores.

Our brief review of investigations suggests that several approaches have been used to study cognition and psychological function in SLE. A majority of studies to date have used self-report questionnaires to classify distress, typically including brief questionnaires that measure depression, anxiety, or general psychological well-being [5,8,11,12,20]. Other studies have attempted to use structured clinical interviews [10,12,15].

Some of the discrepancies in relationships between cognition and depression in SLE may be related to the research methods used to diagnose depression. For example, most studies relating cognition and SLE use standardized measures of depression as noted above. Previously, we reported significant differences between data obtained from participant self-report on standardized questionnaires, physician ratings of depression, and structured psychiatric interviews [23]. A clinical design that minimizes methodological error by classifying major depression by means of structured clinical psychiatric interviews is more likely to accurately define subjects prior to evaluation of cognition. Additionally, there is evidence that depressive symptoms and a diagnosis of major depressive disorder impact aspects of cognitive function, particularly arousal, attention, perception, and memory [24,25]; therefore, studies that examine the similarities and differences in cognitive deficits between participants with only major depressive disorder and depressed SLE participants may yield important information.

Only one such study of depressed SLE and depressed outpatients has been published to date. Denburg and Denburg [26] reported data on 11 patients with SLE and the presence of major depressive disorder (Depressed-SLE), eight depressed psychiatric outpatients, and seven non-depressed patients with SLE. Although the two depressed groups reported similar levels of cognitive, affective, and somatic complaints, the Depressed-SLE patients were more impaired than the depressed outpatients and non-depressed SLE patients in tests of sustained mental effort, verbal and nonverbal learning, and visuospatial planning. This study did not include a control group, so the impact of cognition in the depressed outpatients remains unknown and limits aspects of interpretation. Continued studies in this area are necessary to elucidate the underlying processes of depression and its relationship to neuropsychiatric changes and cognition in SLE.

Our present study compared the performance of Depressed-SLE subjects to Depressed-Controls (history or presence of major depressive disorder only) as well as Healthy Controls on the brief American College of Rheumatology Neuropsychological Battery for SLE (ACR-SLE battery). Additionally, the validity of the ACR-SLE battery was compared to a comprehensive battery in Depressed-SLE participants. Finally, associations between performance on the ACR-SLE battery and measures of depression, fatigue, pain, and perceived cognitive failures were investigated.

## Materials and methods

### Subjects

Participants in this study included 13 Depressed-SLE participants, 10 subjects with the presence of major depressive disorder (Depressed-Controls), and 25 Healthy Controls. All subjects signed an approved consent form authorized by the Institutional Review Board at the National Jewish Medical and Research Center. The SLE participants were obtained from a pool of SLE outpatients seen at the National Jewish Medical and Research Center, the University of Colorado Hospital, and local rheumatology clinics. The primary physician/rheumatologist also completed a neuropsychiatric checklist indicating the presence or absence of neurological and psychiatric symptoms. SLE subjects with possible neurological damage (head trauma; degenerative, vascular, or metabolic disorder; neoplasm; or toxic exposure), major substance abuse, or major psychopathology prior to their diagnoses of SLE were excluded from the study. Any patients with neurological damage or substance abuse following their SLE diagnosis were also excluded. All SLE participants fulfilled the revised criteria for SLE as defined by the ACR [27] as documented by his or her physician.

All of the subjects were screened by means of a detailed neuromedical interview [28]. This interview included questions regarding prior educational, medical, and neurological background. Details regarding prior mental health history (that is, history of therapy, psychotropic medication use, and diagnosis of mood disorder) were also obtained. Subjects recruited for the Depressed-SLE and Depressed-Control groups completed the Structured Clinical Interview for DSM-IV (*Diagnostic and Statistical Manual of Mental Disorders, Fourth Edition*) Non-Patient Version [29] to determine the presence or absence of current major depression. SLE participants with a current major depressive disorder and no other history of neuropsychiatric disorder, based on the interview and a neuropsychiatric checklist completed by the primary physician, were included in the study. Seventy-nine SLE participants were excluded. Of the 13 Depressed-SLE patients included, 76% had a past history of depression that occurred during their diagnosis. Depressed-Control subjects were outpatients recruited from the University of Colorado Health Sciences Center psychiatric clinic and from the Denver metropolitan area (by newspaper advertisements and brochures distributed in various local psychiatric clinics and offices). A neuropsychiatrist and co-investigator on the study (DBA) was available for consultation and review of inclusion/exclusion criteria. All the Depressed-Controls selected for the study had current major depressive disorder (with or without past major depressive disorder) and had no other neurological, medical, or psychiatric disorders. Following screening, 65 Depressed-Controls were excluded and 10 were included. The Healthy Control group was recruited from the Denver metropolitan area by fliers and newspaper advertisements. This group was screened with the same detailed neuromedical interview described above to exclude subjects with histories of learning problems, mental health history, or medical or neuropsychiatric diagnoses.

Subject demographics and health characteristics are presented in Table 1. There were 9 female and 4 male Depressed-SLE participants, 8 female and 2 male Depressed-Controls, and 23 female and 2 male Healthy Controls. As indicated in Table 1, the groups did not significantly differ in age, education level, gender distribution, or race/ethnicity. SLE disease activity was measured with the SLE Disease Activity Index (SLEDAI) [27], which was obtained from each participant's rheumatologist or primary physician at the time of enrollment (within 2 weeks of neuropsychological testing). The mean SLEDAI score for the Depressed-SLE patients was 7.33 (standard deviation [SD] 7.0; range 0 to 21), a score suggesting mild to moderate disease activity. Based on the SLEDAI scores, 27% had a renal disorder, 36% had a hematologic disorder, 9% had pleuritis, 90% had nonerosive arthritis, 35% had a malar rash, and 64% were photosensitive. This group had a mean length of disease of 11.9 years (SD 12.4 years). Ninety-two percent of the Depressed-SLE patients were taking prednisone with a mean level of 9.0 mg (SD 10.7 mg). Additional medications taken by the Depressed-SLE patients included non-steroidal immunosuppressants (62%), anti-hypertensives (77%), anti-depressants (38%), anti-convulsants (23%), and opiates (23%). Among the Depressed-Controls, medication included anti-depressants (40%) and anti-anxiolytics (40%). None of the Healthy Controls was taking prescribed medications.

**Table 1:** Demographics for Depressed-SLE patients, Depressed-Controls, and Healthy Controls

	Depressed-SLE patients	Depressed-Controls	Healthy Controls	*P *value
Number	13	10	25	
Age (years)	42.5 ± 9.5^a^	42.9 ± 10.5^a^	43.5 ± 11.5^a^	0.966
Education (years)	14.2 ± 2.1^a^	14.7 ± 2.3^a^	15.5 ± 2.0^a^	0.173
Gender (female/male)	9/4	8/2	23/2	0.198
Ethnicity (African-American, Caucasian, Hispanic)	0/12/1	0/9/1	2/23/0	0.403

^a^Data are presented as mean ± standard deviation. Depressed-Controls: control subjects with only a history or presence of major depressive disorder; Depressed-SLE: patients with a diagnosis of systemic lupus erythematosus and the presence of major depressive disorder

### Measures

The analyses for this study used a comprehensive cognitive battery (CB), the previously described ACR-SLE battery [28], and questionnaires of depression, pain, fatigue, and perceived cognitive ability.

#### American College of Rheumatology Neuropsychology Battery for SLE

A test battery proposed by the ACR for SLE [30] was administered by a trained neuropsychological technician. The following tests and scores were used in the analyses: Wechsler Adult Intelligence Scale (WAIS)-Revised Digit Symbol Test (total number) [31], Trail Making Test-Part B (total time) [32], Stroop Color and Word Test (Color-Word score) [33], California Verbal Learning Test II (trials 1 to 5 total and short-delay free-recall total) [34], Rey-Osterrieth Complex Figure Test (immediate recall and delayed recall score) [35], WAIS-III Letter Number Sequencing (total score) [36], Controlled Oral Word Association Test and Animal Naming Tests (total scores each) [37], and the Finger Tapping Test (dominant and non-dominant hands) [32]. Reliability and validity for this test battery have been demonstrated [28] by comparison to a larger battery and by test-retest analysis. In addition, a cognitive impairment index (CII) can be calculating using the 12 selected test scores. Each score was converted to *t *scores using demographically corrected normative data, and *t *scores below 40 were considered impaired. The CII has a range of 0 to 12, with a higher number representing greater cognitive impairment [28]. Subjects with four or more out of the 12 *t *scores below 40 were considered cognitively impaired.

#### Comprehensive Neuropsychological Battery

The Comprehensive Neuropsychological Battery (CB), a comprehensive battery previously established as reliable with SLE participants, was administered [5]. The neuropsychological tests evaluate eight cognitive domains: intelligence [31], attention [38,39], reasoning [32], learning [34,40], recall [40], fluency [37,41], language [42,43], and perceptual motor skills [31]. Subjects with two or more out of eight cognitive domains below a mean *t *score of 40 were classified as cognitively impaired. In our initial study, approximately 33% of the non-NPSLE participants and 11% of the controls were classified as impaired based on this criterion [5].

#### Center for Epidemiological Studies Depression Scale

The Center for Epidemiological Studies Depression Scale (CES-D) [44] is a self-administered 20-item questionnaire that measures the subject's state on a scale of 0 ('rarely or none of the time') to 3 ('most or all of the time') with regard to mood and vegetative motor functions during the preceding week. Total scores range from 0 to 60. A score of 16 or higher indicates symptoms consistent with clinical depression [44]. This scale has demonstrated adequate reliability and validity in various settings and across ethnic backgrounds. A total CES-D score was calculated.

#### Multidimensional Assessment of Fatigue Questionnaire-Modified

The Multidimensional Assessment of Fatigue Questionnaire-Modified (MAF) [45] is a self-administered 16-item questionnaire that assesses four dimensions of fatigue over the past week: severity (items 1 to 3), impact on activities of daily living (items 4 to 14), and timing (items 15 and 16). The first 14 items are rated on a scale of 1 ('not at all') to 10 ('a great deal'). A global fatigue scale is calculated from the total.

#### Short-Form McGill Pain Questionnaire

The Short-Form McGill Pain Questionnaire (MPQ) [46] provides a qualitative and quantitative assessment of pain. It contains 15 pain-related words divided into sensory and affective categories in a pain-rating index. Individuals are asked to give each description of pain a rating from 0 ('none') to 3 ('severe') based on the degree to which he or she feels that type of pain. Total pain is calculated by adding up these ratings. The range is from 0 to 45 and is labeled McGill Pain Total.

#### Cognitive Failures Questionnaire

The Cognitive Failures Questionnaire (CFQ) [47] is a self-administered 25-item questionnaire that measures everyday cognitive errors of attention, perception, memory, and motor functioning over the past 6 months on a 5-point scale that ranges from 0 ('never') to 4 ('very often'). Total scores for the CFQ range from 0 to 100.

### Statistical analysis

All statistical analyses were conducted with the SAS statistical analysis package (version 9.1; SAS Institute Inc., Cary, NC, USA). Data are presented as means ± SDs for continuous variables and as numbers of subjects for categorical variables. An analysis of variance (ANOVA) model and Fisher exact test were used to evaluate the overall group differences in demographic variables. An ANOVA model was also used to compare neuropsychological tests and measures of depression, fatigue, pain, and perceived cognitive deficits between the three groups. In this ANOVA model, each test score entered the model separately as the outcome variable and group was used as the predictor variable. Post hoc analyses were performed using the Tukey-Kramer multiple comparisons procedure. Intra-class correlation coefficients (r) were calculated via a one-way random-effects ANOVA model. A simple linear regression model was used to assess the association between CII and measures of fatigue, depression, pain, and perceived cognitive deficits; A cognitive impairment index (ACR-SLE-CII or CB-CII) was used as the outcome variable and one of the above measures was used as the predictor variable. For all of the analyses, *p *values less than 0.05 were designated to be statistically significant.

## Results

### Comparison of ACR-SLE battery subtests across groups

The ACR-SLE battery includes 10 tests with a total of 12 scales. Each scale was demographically corrected using available normative data for each test to calculate a *t *score [34]. The mean *t *score and SD for each test by group are presented in Table 2. The *t *scores were compared between groups for each of the 12 scaled scores using a one-way ANOVA model. As indicated, scores were significantly different across groups for three tests: the Digit Symbol Test, the Stroop Color-Word Score, and the Trail Making Test-Part B. Post hoc analyses were performed using the Tukey-Kramer multiple comparisons procedure, with the experiment-wise type I error rate at the 5% level. Results illustrated in Table 2 indicate that the Depressed-SLE patients differed from Healthy Controls on the Digit Symbol Test, the Stroop Color-Word Score, and the Trail Making Test-Part B. The Depressed-Controls differed from Healthy Controls on the Stroop Color-Word Score. The Depressed-SLE participants scored lower on the Digit Symbol Test compared to Depressed-Controls (*p *= 0.049).

**Table 2:** Test scores by group from the American College of Rheumatology neuropsychological battery for patients with systemic lupus erythematosus

	Depressed-SLE patients (1)^a^	Depressed-Controls (2)^a^	Healthy Controls (3)^a^	*P *value	Post hoc analyses
WAIS Revised Digit Symbol Test	43.1 ± 10.7	52.2 ± 8.0	55.3 ± 8.2	0.001	1 < 2, 3
WAIS Third Edition Letter Number Sequencing Test	50.3 ± 9.3	51.0 ± 6.5	50.7 ± 9.7	0.984	
Stroop Color and Word Test Color-Word score	42.5 ± 8.7	41.9 ± 8.4	52.4 ± 7.6	<0.001	1, 2 < 3
Trail Making Test-Part B	42.8 ± 14.7	47.1 ± 9.8	57.9 ± 10.6	0.001	1, 2 < 3
Controlled Oral Word Association Test	48.0 ± 10.3	46.3 ± 8.6	49.1 ± 9.8	0.736	
Animal Naming Test	48.5 ± 8.4	52.4 ± 7.8	53.5 ± 11.4	0.350	
CVLT trials 1–5	48.7 ± 12.1	46.3 ± 6.5	52.0 ± 14.4	0.574	
CVLT short-delay free-recall	48.5 ± 9.0	50.0 ± 12.5	50.7 ± 12.2	0.712	
Rey-Osterrieth Complex Figure Test: immediate recall	41.8 ± 15.7	44.6 ± 9.9	48.0 ± 12.6	0.375	
Rey-Osterrieth Complex Figure Test: 30-minute delayed recall	44.1 ± 15.3	46.6 ± 12.2	47.9 ± 11.4	0.678	
Finger Tapping Test, dominant hand	47.3 ± 16.6	51.0 ± 8.2	54.8 ± 10.6	0.200	
Finger Tapping Test, non-dominant hand	46.8 ± 16.2	51.2 ± 6.6	55.2 ± 11.8	0.143	

^a^Data are presented as mean *t *score ± standard deviation. CVLT: California Verbal Learning Test; Depressed-Controls: control subjects with only a history or presence of major depressive disorder; Depressed-SLE: patients with a diagnosis of systemic lupus erythematosus and the presence of major depressive disorder; WAIS: Wechsler Adult Intelligence Scale.

**Figure 1:**
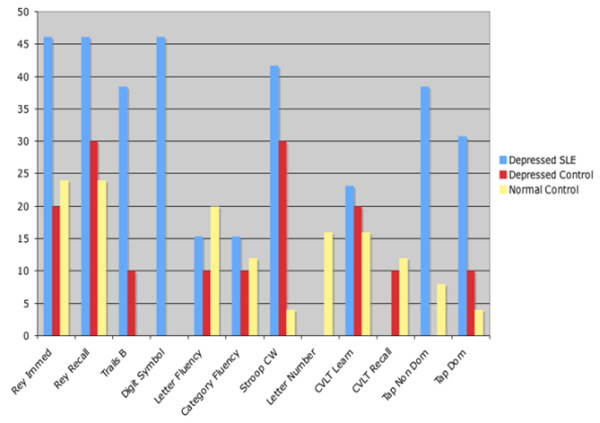
Percentage of impairment on American College of Rheumatology battery tests across groups. Category Fluency: Animal Naming Test; Digit Symbol: Wechsler Adult Intelligence Scale Revised Digit Symbol Subtest; CVLT Learn: California Verbal Learning Test Form II Trials 1–5 Learning; CVLT Recall: California Verbal Learning Test Form II Short-Delay Free Recall; Depressed Control: control subjects with only a history or presence of major depressive disorder; Depressed SLE: patients with a diagnosis of systemic lupus erythematosus and the presence of major depressive disorder; Letter Fluency: Controlled Oral Word Association Test; Letter Number: Wechsler Adult Intelligence Scale-III Letter Number Sequencing subtest; Rey Immed: Rey Osterrieth Complex Figure Test Immediate Recall; Rey Recall: Rey Osterrieth Complex Figure Test Delayed Recall; Stroop CW: Stroop Color Word Test Color-Word score; Tap Dom: Finger Tapping Test (Dominant Hand); Tap Non Dom: Finger Tapping Test (Non-Dominant Hand); Trails B: Trail Making Test-Part B.

Scores below a *t *score of 40 were designated as impaired for all the test scores on the ACR-SLE battery. The frequency of impairment on these tests for the groups is presented in Figure 1. This figure illustrates that the highest frequency of impairment across most of the tests was in the Depressed-SLE group; more than 40% of these subjects were impaired on the Digit Symbol total number, Stroop Color-Word total score, Rey-O learning score, and Rey-O delayed recall score; more than 30% were impaired on the Trail Making Test-Part B and Finger Tapping bilaterally.

### Comparison of comprehensive domain and subtests between groups

As indicated in Table 3, the three groups differed significantly across domain scores of reasoning, attention, recall, and perception. Depressed-SLE participants performed worse than Healthy Controls on all of these domains in post hoc analyses. Depressed-Controls performed worse than Healthy Controls on reasoning. A trend for lower scores on perception was noted in the Depressed-SLE group compared to Depressed-Controls (*p *= 0.056). In terms of specific subtests with overall group differences, the Depressed-SLE group performed worse than Healthy Controls on Performance Intelligence Quotient, Trail Making Test-Part A, PASAT (Paced Auditory Serial Addition Test), Story Learning, Story Recall, and Block Design. The Depressed-Controls performed worse than Healthy Controls on Trail Making Test-Part A. The Depressed-SLE participants performed worse than Depressed-Controls on Story Learning and Object Assembly. Finally, a trend for lower scores on Story Recall was noted in the Depressed-SLE group compared to Depressed-Controls (*p *= 0.08)

**Table 3:** Comparison of individual and domain neuropsychology test scores from the comprehensive battery between groups

Variable	Depressed-SLE patients (1)^a^	Depressed-Controls (2)^a^	Healthy Controls (3)^a^	*P *value	Post hoc analyses
Intelligence	45.5 ± 9.4	49.8 ± 6.9	51.5 ± 6.8	0.084	
WAIS-R Verbal IQ	47.2 ± 10.6	48.0 ± 8.1	49.8 ± 7.7	0.645	
WAIS-R Performance IQ	43.9 ± 10.3	51.5 ± 9.1	53.2 ± 7.7	0.012	1 < 3
Reasoning	45.7 ± 11.0	47.0 ± 5.6	55.7 ± 6.2	<0.001	1, 2 < 3
WAIS-R Similarities	51.2 ± 9.6	48.6 ± 7.3	51.4 ± 9.6	0.703	
Category Test	46.8 ± 17.3	44.2 ± 6.6	52.2 ± 9.3	0.141	
Trail Making Test-Part A	42.1 ± 13.0	48.2 ± 9.7	61.2 ± 9.5	<0.0001	1, 2 < 3
Attention	41.8 ± 7.2	44.5 ± 5.4	50.0 ± 6.9	0.002	1 < 3
Digit Vigilance Test-Time	44.7 ± 14.2	54.8 ± 11.9	54.8 ± 11.6	0.051	
Digit Vigilance Test-Errors	48.0 ± 11.9	38.1 ± 13.2	48.1 ± 12.0	0.085	
PASAT total	32.7 ± 15.7	41.7 ± 10.2	47.2 ± 9.0	0.002	1 < 3
Learning	42.0 ± 7.3	47.2 ± 6.6	47.5 ± 7.9	0.096	
Figure learning	41.5 ± 8.2	47.7 ± 10.3	46.7 ± 13.7	0.354	
Story learning	35.8 ± 6.8	47.6 ± 9.5	45.2 ± 9.2	0.003	1 < 3; 1 < 2
Recall	44.1 ± 7.6	49.1 ± 7.3	51.3 ± 7.2	0.021	1< 3
Figure recall	48.8 ± 9.6	49.1 ± 9.3	53.8 ± 6.4	0. 120	
Story recall	39.3 ± 12.4	49.0 ± 9.2	48.9 ± 10.1	0.028	1< 2, 3
Fluency	49.5 ± 7.4	47.7 ± 4.1	48.2 ± 6.5	0.775	
Figural fluency	47.4 ± 10.4	44.9 ± 13.2	47.3 ± 8.8	0.814	
Language	46.9 ± 6.3	51.2 ± 9.0	48.4 ± 7.0	0.378	
Oral-verbal comprehension	46.1 ± 9.3	51.2 ± 7.7	47.5 ± 7.9	0.327	
Written comprehension	47.8 ± 11.9	51.2 ± 13.6	49.4 ± 11.4	0.793	
Perception	43.6 ± 11.5	52.8 ± 9.2	52.6 ± 7.4	0.017	1 < 3
WAIS-R Block design	45.5 ± 11.7	51.0 ± 10.5	56.2 ± 10.2	0.018	1 < 3
WAIS-R Object assembly	41.7 ± 12.3	54.5 ± 10.7	48.9 ± 7.6	0.011	1<2

^a^Data are presented as mean ± standard deviation. Depressed-Controls: control subjects with only a history or presence of major depressive disorder; Depressed-SLE: patients with a diagnosis of systemic lupus erythematosus and the presence of major depressive disorder; Figural fluency: Ruff Figural Fluency Test; Figure learning: Learning component of Figure Memory Test; Figure recall: Delayed component of Figure Memory Test; IQ: Intelligence Quotient; Letter fluency: Controlled Oral Word Association Test; Oral-verbal comprehension: Boston Diagnostic Aphasia Examination-Complex Ideational Material Test; PASAT: Paced Auditory Serial Addition Test; Story learning: Learning component of Story Memory Test; Story recall: Delayed component of Story Memory Test; WAIS-R: Wechsler Adult Intelligence Scale-Revised; Written comprehension: Peabody Individual Achievement Test reading recognition test.

### Cognitive impairment indices between groups

We compared the CII from the ACR-SLE battery (ACR-SLE-CII), and found significant differences between groups (*p *= 0.015). The Depressed-SLE group had a mean ACR-SLE-CII of 3.4 (SD 2.4), Depressed-Controls had a mean of 1.5 (SD 1.5), and Healthy Controls had a mean of 1.6 (SD 1.6). Post hoc analyses using the Tukey-Kramer method adjusting for multiple comparisons indicated that the Depressed-SLE participants had a higher ACR-SLE-CII compared to the Depressed-Controls (*p *= 0.048) and Healthy Controls (*p *= 0.018). A significant group difference was also noted across the CB-CII (*p *= 0.01); post hoc analyses indicated that the Depressed-SLE participants had a greater CB-CII compared to the Depressed-Controls (*p *= 0.048) and Healthy Controls (*p *= 0.012).

### Agreement between the ACR-SLE and CB neuropsychological batteries

Overall levels of impairment on the ACR-SLE and CB batteries were computed based on prior methods [28]. Sensitivity (se) and specificity (sp) were calculated for the ACR-SLE battery compared to the CB, and a kappa statistic (κ) was calculated as a measure of agreement between the batteries. The ACR-SLE-CII had an overall agreement of 90% (se = 80%, sp = 92.1%, κ = 0.70) with the original comprehensive battery impairment, suggesting almost perfect agreement. In the Depressed-SLE group, agreement between the two batteries was 84.6% (se = 66.7%, sp = 100%, κ = 0.68) with 6/13 (46%) impaired on the comprehensive battery and 4/13 (30%) impaired on the ACR-SLE battery. This level suggested moderate agreement between the two batteries in the Depressed-SLE group. In the Depressed-Control group, agreement between the two batteries was 80% (se = 50%, sp = 87.5%, κ = 0.38) with 2/10 (20%) of the participants impaired on the comprehensive battery and 2/10 (20%) impaired on the ACR-SLE battery. This suggests marginal agreement between the two batteries in the Depressed-Controls. In the Healthy Control group, agreement between the two batteries was 96% (se = 75%, sp = 100%, κ = 0.83) with 3/25 (12%) impaired on the comprehensive battery and 4/25 (16%) impaired on the ACR-SLE battery, which indicates excellent agreement between the two batteries.

### Relationship between ACR-SLE tests and measures of depression, fatigue, pain, perceived cognitive deficits, and health variables

Both the Depressed-SLE group and the Depressed-Controls had higher scores compared to Healthy Controls (*p *< 0.001) on all self-reported scales of depressive symptoms (CES-D), pain (MPQ), fatigue (MAF), and cognitive failures (CFQ). The Depressed-SLE group and Depressed-Controls did not differ in terms of CES-D total (29.6 versus 30.7), MAF Global Fatigue (38.0 versus 32.2), or CFQ (60.0 versus 55.8). The Depressed-SLE patients did have higher overall pain on the MPQ (mean 18.5, SD 1.9) compared to the Depressed-Controls (mean 10.9, SD 2.1) (*p *= 0.03).

Using simple linear regression models, we also evaluated the relationship between (a) ACR-SLE-CII and CB-CII and (b) measures of fatigue (MAF), depression (CES-D), pain (MPQ), and perceived cognitive deficits (CFQ). When the groups were analyzed separately, we found no associations between (a) ACR-SLE-CII and CB-CII and (b) CES-D, MPQ, MAF, and CFQ.

For the Depressed-SLE group, no associations were reported between the individual ACR-SLE battery tests and SLEDAI score total and length of diagnosis. Only one of the measures (category fluency) was associated with prednisone use, indicating that those on prednisone had a lower fluency score (*p *= 0.02); however, none of the other 11 test scores showed an association.

## Discussion

The results in this study indicate that the Depressed-SLE group performed worse than the Depressed-Controls and Healthy Controls on a cognitive impairment index, a global score of cognitive functioning. These findings are consistent with preliminary work done by Denburg and Denburg [26], suggesting that patients with SLE and depression are more cognitively impaired than depressed outpatients without SLE. The Depressed-SLE group was specifically impaired compared to our Healthy Controls on three measures of attention, four measures of visuomotor speed, one measure of visuoconstructive abilities, and a measure of learning and memory for story-like information. In comparison, the Depressed-Controls performed worse than the Healthy Controls on one measure of attention, one measure of visuomotor speed, and one overall reasoning domain. In general, the Depressed-Controls had fewer overall deficits in cognitive testing than did the Depressed-SLE group, suggesting that depression in SLE has a different effect on cerebral function than depression alone.

The Depressed-SLE patients performed more poorly on measures of verbal learning, visual motor functions, and visuomotor speed compared to the Depressed-Controls. This observation appears to confirm a pattern of visuospatial deficits and attention problems noted by Denburg and Denburg [26]. Cognitive impairment in specific neuropsychological domains (that is, attention, memory, and executive functions) may be related to abnormalities in the specific neuroanatomic regions in Depressed-SLE participants compared to Depressed-Controls. Notably, none of the SLE disease variables (that is, disease activity, length of disease, or prednisone use) was correlated with impairment on these cognitive measures.

In this study, Depressed-SLE participants showed specific deficits in learning and recall for verbal material when compared to Depressed-Controls. Specific deficits of verbal and nonverbal learning and memory have frequently been reported in SLE [5,8,12,16,18,48]; the hippocampus has been identified as a potential region of interest in this population given its relationship to memory. Recently, SLE patients with elevated levels of anti-*N*-methyl-D-aspartate (NMDA) (an autoantibody associated with hippocampal damage) had poor performance on measures of immediate memory, fine motor function, and psychological functioning [49]. This observation offers at least one possible mechanism by which SLE, and not idiopathic major depressive disorder, may cause impairments in learning and recall for verbal material: autoimmune-mediated injury to the hippocampus, a requisite structure for the process of forming new declarative memories.

This study's Depressed-SLE participants had a greater frequency of attentional deficits compared to Healthy Controls and Depressed-Controls. Frontal white matter abnormalities are another region of interest in SLE given this neuroanatomic region's relationship to attention and efficiency of information processing [50]. In our previous work, non-NPSLE participants were found to have increased white matter hyperintensities that were associated with attentional deficits [51]. These findings suggest that cognitive changes in patients with SLE may relate to subtle changes in white matter. In a subsequent study [53] using magnetic resonance spectroscopy, elevations of choline/creatine levels, a neurobetabolic measure of inflammation, were related to increased cognitive impairment in non-NPSLE participants. Several studies suggest that white matter deterioration occurs in SLE [52], and damage to cerebral white matter may be a neuropathological event that has cognitive consequences in Depressed-SLE participants.

Correlations between the cognitive impairment in Depressed-SLE participants and measures of fatigue, pain, and perceived deficits were not found. Interestingly, in a prior study with a diverse group of NPSLE participants, we found strong correlations between cognitive dysfunction and fatigue, pain, depression, and perceived dysfunction [11]. The lack of a relationship between behavioral measures may relate to the modest sample size and lack of power to detect associations. Conversely, it may suggest that pain and fatigue are not significantly responsible for the cognitive impairment seen in the current sample of Depressed-SLE patients.

Psychometric properties of the brief ACR-SLE battery and the CB were evaluated and indicate that the ACR-SLE battery is valid for research in depressed participants with SLE. In our prior study, we noted that associations between the brief ACR-SLE battery and the CB are 95% for non-NPSLE and 81% for patients with a previous history of NPSLE [28]. This study shows that agreement between the two batteries remains reliable at 84.6% in a subgroup of SLE participants with current depression (Depressed-SLE).

Some methodological issues limit conclusions from this study. For example, the small sample size of our depressed groups reduced the overall conclusions and general applications of our findings. It also limited our statistical capabilities and, as noted earlier, may have suppressed some potential findings. Notably, of the 92 SLE participants screened, only 14% had major depression without other neuropsychiatric disorders. This suggests that this subgroup of patients with SLE is a very select group of patients, and that continued investigations in such a group may improve scientific understanding of depression in SLE.

## Conclusion

Despite methodological limitations, our findings indicate that Depressed-SLE participants have greater cognitive deficits compared to Depressed-Controls and Healthy Controls. Specific deficits in learning and attentional skills were more frequent in the Depressed-SLE group compared to Depressed-Controls. Notably, there were several tests impaired in both of our depressed groups, indicating that depression is a common contributing factor. This study does not allow investigation into the processes by which Depressed-SLE patients had greater deficits, and it remains unclear if these processes are biologically mediated and specific to SLE or if they are related to chronic disease in general. Continued studies investigating immunological (that is, NMDA) and neuropathological (that is, neurometabolite functioning and quantitative brain morphology) abnormalities in this type of SLE patients are necessary to understand biological mechanisms related to neurobehavioral changes in this population. Clinically, this suggests that cognitive concerns in patients with SLE and depression may require additional evaluation by neurology, neuropsychiatry, and/or neuropsychology specialists.

## Abbreviations

ACR = American College of Rheumatology; ACR-SLE battery = American College of Rheumatology Neuropsychological Battery for systemic lupus erythematosus; ACR-SLE-CII = American College of Rheumatology Neuropsychological Battery for systemic lupus erythematosus cognitive impairment index; ANOVA = analysis of variance; CB = Comprehensive Neuropsychological Battery; CB-CII = Comprehensive Battery cognitive impairment index; CES-D = Center for Epidemiological Studies Depression Scale; CFQ = Cognitive Failures Questionnaire; CII = cognitive impairment index; CNS = central nervous system; Depressed-Control = control subject with only a history or presence of major depressive disorder; Depressed-SLE = patient with a diagnosis of systemic lupus erythematosus and the presence of major depressive disorder; κ = kappa statistic; MAF = Multidimensional Assessment of Fatigue Questionnaire-Modified; MPQ = McGill Pain Questionnaire; NMDA = *N*-methyl-D-aspartate; non-NPSLE = systemic lupus erythematosus with inactive disease and without overt neuropsychiatric disorders; NPSLE = systemic lupus erythematosus with overt neuropsychiatric disorders; SD = standard deviation; se = sensitivity; SLE = systemic lupus erythematosus; SLEDAI = Systemic Lupus Erythematosus Disease Activity Index; sp = specificity; WAIS = Wechsler Adult Intelligence Scale.

## Competing interests

The authors declare that they have no competing interests.

## Authors' contributions

EK was the principal investigator of the study and the primary author of the manuscript. She was responsible for writing the initial research design, supervising subject enrollment, protocol administration, data collection, and interpretation of data. DBA was a co-investigator on the study and contributed to the initial study design, recruitment of depressed control subjects, and inclusion/exclusion of depressed SLE patients. He made a major contribution to manuscript preparation and to the discussion of results. LZ was the biostatistician responsible for all the statistical analyses presented in the paper. She also made major contributions to the manuscript with regard to statistical procedures. SW was a co-investigator on the study, was responsible for the accurate inclusion/exclusion of SLE patients, and reviewed neuropsychiatric criteria and SLEDAI activity. He also contributed to the manuscript preparation and discussion of results. All authors read and approved the final manuscript.
